# Habronematidosis in Equids: Current Status, Advances, Future Challenges

**DOI:** 10.3389/fvets.2020.00358

**Published:** 2020-07-03

**Authors:** Alessandra Barlaam, Donato Traversa, Roberto Papini, Annunziata Giangaspero

**Affiliations:** ^1^Department of Science of Agriculture, Food and Environment, University of Foggia, Foggia, Italy; ^2^Faculty of Veterinary Medicine, University of Teramo, Teramo, Italy; ^3^Department of Veterinary Science, University of Pisa, Pisa, Italy

**Keywords:** habronematidosis, epizootiology, clinical signs, diagnosis, control

## Abstract

Over the past few decades, among equine parasitoses caused by gastrointestinal nematodes, habronematidosis has been discontinuously studied worldwide. Habronematidosis is a parasitic disease distributed all over the world. It is caused by *Habronema microstoma, Habronema muscae*, and *Draschia megastoma* (Spirurida, Habronematidae), and it is maintained in the environment by muscid flies which act as intermediate hosts. At larval and adult stages these species live in the stomach of domestic and wild equids. However, the larvae can also be found on the skin, causing lesions known as “summer sores”, and occasionally on other body areas, such as ocular and genital mucosa (muco-cutaneous habronematidosis) and lung, liver, brain parenchyma. Depending on the parasite's developmental stage and localization site, clinical signs vary from mild to severe. Habronematidosis is responsible for significant economic losses, mostly when sport horses are affected, because their performances are impaired and the infection can be unaesthetic. We used three on-line databases for searching the articles on habronematidosis according to the selected inclusion criteria; a total of 250 contributions, published between 1911 and 2020 were analyzed. This review summarizes the key features of pathogenesis, epizootiology, diagnosis, treatment, and control of habronematidosis, and highlights the current knownledge about its geographical distribution and spread. Anthelmintic drugs are the most widely-used tools against habronematidosis; given the known risk of anthelmintic resistance in some nematodes affecting horses, this aspect should also be explored for habronematidosis. Dedicated research is essential to fill gaps of knowledge and increase the understanding of habronematidosis to maximize equine health, reduce economic losses and sanitary impact associated with this parasitic infection.

## Introduction

The fascinating history of habronemiasis—from now on called habronematidosis—dates back to the second decade of 1900, when in an interesting Special Article published on *Science*, Ransom (1), a bright zoologist of the Bureau of Animal Industry, in Washington DC, states: “*Fifty years ago, from Bombay, India, the late H. J. Carter reported the discovery of nematodes parasitic in the house fly, giving them the name of* Filaria muscae.”

This means that the fortune of this parasite derived from its fortuitous discovery not in the animal victim but in its intermediate host. In fact, when in the summer of 1911, “*a series of stages in the development of the parasite was obtained by examination of various stages of the fly from larva to imago*” (…), the hypothesis that “Habronema muscae *is the larval stage of a nematode parasitic during its adult stage in (….) the stomach of the horse*” was postulated. This was confirmed soon later when in a few horses “*examined shortly after death*” (…), not only “*a few adult nematodes were found*” but also “*a complete series of stages in the development and growth of a single species of nematode from larva to adult*”.

Indeed, the knowledge of the entire life-cycle of *Habronema* was described thanks to the results of those old and pioneering investigations.

Since that intense period of studies, habronematidosis has been intermittently studied with none or a few original and on the field studies up to the ‘80s. In this decade, the knowledge of this parasitosis had a significant impulse following the discovery (in the late-1970s) of the innovative drug ivermectin (2), introduced commercially in 1981. More often, these studies on habronematidosis coincided with specific investigations on the gastrointestinal nematodofauna (from prevalence to pathological aspects) in slaughtered horses finalized later on to the control of these parasites. Afterward, only a few studies dealt specifically with habronematidosis until the attention to sport horses highlighted the importance of the “summer sores” in these valuable animals and the need of controlling gastric forms of these spirurids in the definitive hosts. Thereafter, the advent of molecular tools opened new interesting scenarios for the comprehension of this widespread—but still not completely understood—intriguing parasitic disease.

Here, a broad review of habronematidosis, covering all aspects (etiology, epizootiology, clinics, pathology, diagnosis, prophylaxis, and therapy) were undertaken. In addition, key weaknesses and knowledge gaps were identified and key suggestions for future research were provided. Thus, we scrutinized 250 articles/books published between May 1911, and April 2020, with no language restrictions^1^.

## Etiology and Life Cycle

Among the 12 species of *Habronema* listed as parasites of mammals (3), *Habronema microstoma* (syn. *Habronema majus*^2^)*, Habronema muscae*, and *Draschia megastoma* (former *Habronema megastoma*) (Spirurida, Habronematidae) are the only ones detected in domestic (horses, donkeys, mules) and wild equids (zebras).

*Habronema microstoma* is a whitish worm (female and male, 15–35 and 9–22 mm of length, respectively), narrowed slightly at the anterior end, with a single lateral ala. The buccal vestibule is greatly thickened and has two tridentate teeth (5). The pharynx is cylindrical and provided with a dorsal and a ventral tooth, called “pharynx teeth” (5, 6).

*Habronema muscae*, closely resembles *H. microstoma*; the differences concern the color as adult (yellow pale or orange) and the pharynx, which is not provided with teeth.

*Draschia megastoma* adults (7–13 mm long) are white and their head is separated from the rest of their body by a visible constriction. The pharynx is funnel-like, with two separated lateral vales; no teeth are present (7).

The adults of all three species (*Habronema muscae, H. microstoma* and *D. megastoma*) live in the stomach wall of the gastric fundus and pyloric valve or freely on the mucosal surface of the *margo plicatus* (8). After mating, females release eggs (40–80 μ × 10–20 μ in size—with *H. muscae* reaching the biggest size), elongate in *Habronema* and cylindrical in *Draschia*, containing larvae, which may either hatch during intestinal transit or in the environment after release via feces. The first stage larvae (L1) are motile and show a positive hydrotropism and thermotropism; they can live as long as 7 days under suitable environmental conditions. Eggs and/or larvae are then ingested by dung-inhabiting muscid larvae. *Musca domestica* and *Stomoxys calcitrans* are the main vectors of *H. muscae* and *H. microstoma*, respectively. The larvae and the insect develop synchronously. In fact, *H. microstoma* and *H. muscae* develop further at about a similar time as the fly *imago* emerges from the *puparium* (1, 9).

The larval development of *H. muscae* in *M. domestica* in laboratory conditions has been nicely described by Amado et al. (10). Three to 5 days post infection, *Habronema* L1 were found free in the hemocoel and in fat cells of muscid L3. From 4th to 7th days p.i. two morphotypes of L2, robust and elongate, respectively, can be simultaneously present. The first is located in intracellular fat cell-like structures whereas the elongate form is located into capsules formed by syncytial tissue; the robust type was recovered from fly larvae post-feeding whereas both morphotypes from cryptocephalic pupae, pupae and pharate adults. *Habronema* L3 were found in thin and elastic capsules inside the mature pupae and adults, fixed to different fly organs, including middle and final intestine. From 48 h post-emergence L3 reach the fly head (10).

Stimulated by the warmth, *Habronema* L3 are deposited by the flies around the animals' lips; horses swallow them and the larvae develop into adults in the animals' stomach, causing the gastric form. When the larvae are deposited on other cutaneous sites (cutaneous form) or eyes, nostrils, genital mucosa (muco-cutaneous form), or when, as rarely occur, they reach the lungs (pulmonary form), liver, brain (erratic form) (11), they do not achieve sexual maturity.

## Epizootiology

Habronematidosis is distributed worldwide mostly in tropical and subtropical areas, but it is also prevalent (enzootic) in temperate regions, including the Mediterranean countries (12). Prevalence differs significantly among countries and data comparison was difficult due to the limitations and large differences in the study designs. Taking into account only medium/large-scale epizoological investigations, gastric habronematidosis has been reported in Europe affecting 1.1% of horses in Sweden (13), 4.3% in The Netherlands (14), 8.5% in France (15), 17% in Belgium (16), roughly 20% in Poland (17), 33% in Germany (18). In North America the prevalence ranges from 11 to 62% (19), whereas in Australia it reaches peaks of 72% (20, 21). In Africa there is the highest infection prevalence: 62–100% of donkeys and/or horses were found positive (22–27).

Up to 2,000 individual parasites (mean 500) have been counted in a single animal stomach (28); however, in some areas (i.e., Morocco), up to 4,000 individuals have been detected (23).

Although cutaneous or muco-cutaneous habronematidosis are described, especially in temperate regions (29), the prevalence of these forms is lacking, mainly because of clinical diagnosis limitations. The description of these cases is often limited to single cases, for instance in UK (30, 31), Belgium (32), and Italy (12). An increase of (peri)ocular habronematidosis has been recently suspected in the Netherlands (33).

Although the responsible for the infestation is often unidentified, both in gastric and cutaneous forms, when identifications occur, regardless the forms, *H. muscae* is the most detected species. *Draschia megastoma*—originally described from horses in Germany (34)—is currently considered a rare parasite (17, 18, 35–37). It is a frequent species in the USA where the percentage of positive animals ranges from 24 to 62% (19, 28, 38) or in Australia where the infection rate varies from 39 to 41% (20, 21).

For both gastric and cutaneous habronematidosis, the infection does not seem to be age dependent (39).

Although several fly genera of Diptera (Muscidae) (*Musca, Fannia, Sarcophaga, Haematobia, S*. *calcitrans*) have been incriminated as possible vectors of habronematidosis in field conditions, only *S. calcitrans* and *M. domestica* have been proven to transmit *H. microstoma* and *H. muscae*, respectively (40). These two species are the most closely associated with the environments where horses are kept. Larval stages of both species are dung-inhabitants; adults of *M. domestica* (secretophagous) feed on eyes, nose and mouth of the host, whereas *S. calcitrans* are blood-feeding, and attack the animals mostly on their legs and flanks.

Interestingly, it has been recently shown that (i) longer is the exposition of *M. domestica* to *H. muscae*, higher is the average larval burden of *H. muscae* in the emerged flies; (ii) the proportion of insect larvae that develop into adults is lower in infected groups; (iii) in infected groups pupae are smaller and lighter. Whether this is attributable to the destruction of adipose cells in the maggots by *Habronema* larvae or not, requires a more in-depth investigation (41).

The seasonality of the intermediate hosts influences the seasonal trend of habronematidosis. In temperate climates, the infection reaches its peak in summer; in tropical areas the spread of *H. microstoma* reaches high levels in January and in July-September, while *H. muscae* especially in January-March (23); it is therefore conceivable that, at least in some regions, the two *Habronema* species have “preferential” species of flies as intermediate hosts.

It is interesting to point out that, whilst the role of *M. domestica* as a vector of *H. microstoma* remains to be better investigated (40, 42), the host-parasite association between *M. domestica* and *H. muscae* appears more biologically and developmentally settled. This seems to be related to the ability of *M. domestica* to stabilize *H. muscae*; in fact, although there is an inverse relation between the intensity of infection by *H. muscae* and the longevity of *M. domestica*, a low level of infestation does not interfere with the dipterous reproduction and consequently guarantees the maintenance of habronematidosis (43). This aspect confirms the adaptation process related to coevolutive processes. Furthermore, the presence of ultrastructural “anatomical devices” on *H. muscae* infective L3 seems to help them in the rupture of the muscoid proboscis and in the movements to reach the horse (10). At the same time, it cannot be excluded that the strong ability of *M. domestica* in transmitting *H. muscae* may be related to the high number of infected houseflies in horse farms together with a high prevalence and mean intensity of *H. muscae* infection in horses (10).

## Clinical Signs and Histopathological Aspects

Clinical signs related to *H. microstoma, H. muscae*, and *D. megastoma* infection depend on the parasite's stage of development and localization. Adults have a double effect on the host: a mechanical-irritative and a toxic effect, caused by the metabolites they produce.

At least four clinical forms of habronematidosis are known, according to the localization of the nematodes.

In the *gastric habronematidosis, Habronema*, and/or *Draschia* are confined at the level of gastric mucosa glands and responsible of different degrees of atrophy, mechanical irritation of the stomach, secretory and functional disorders; clinical signs can range from no signs to anorexia/dysorexia, digestive disorders, diarrhea, gradual weight loss; also they may predispose horses to ulcers and postprandial colics (44, 45). Adults of *D. megastoma* create large swellings, which may hamper the peristalsis of the stomach, or impede the pyloric opening. This nematode occasionally causes acute hemorrhages or damage of the stomach wall leading to acute peritonitis and even death (46, 47). There are often congested and hemorrhagic ulcer-like areas, which can be isolated or confluent, especially when *H. microstoma* is present. *D. megastoma* causes granulomatous lesions with central necrosis, cellular debris and eosinophilic infiltration. When *H. microstoma* and/or *H. muscae* is present, an abundant secretion envelops the parasites. A close agreement between the number of infected horses by *D. megastoma* and the presence of lesions have been also noticed (19).

In the stomach of affected donkeys, at necropsy hyperaemia, erosions and ulcers, oedema, together with parasitic lesions are visible. Ulceration of the non-glandular gastric regions is more prominent than the glandular regions (48). Histologically, hyperkeratosis, acanthosis, vacuolar degeneration of squamous cells, erosions, ulcerations, hyperfunction of mucus glands have been described in donkeys (48).

**Figure 1:**
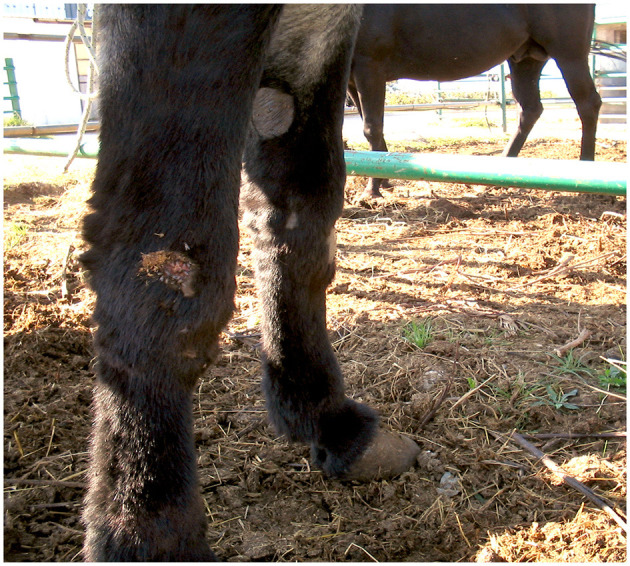
Cutaneous habronemosis in a donkey (original, D. Traversa).

The *cutaneous habronematidosis* is the most severe form, and lesions are known as “summer sores” (Figure 1). Wounds tend to disappear spontaneously in the cold months but re-appear when the environmental temperature rises again months later. It is still unclear whether in winter the larvae remain in the lesions in a dormant state, and reactivate later in the following warm season or not (49). L3 are deposited on the wounds by flies and the spine at the larval posterior end is responsible for the injury and for the local hypersensitivity reaction. Chest, fetlocks and the inner side of the legs are the most affected body areas.

Clinical signs range depending on the origin/time of the lesion. Skin lesions may be single or multiple and are proliferative, exuberant and granulomatous, frequently bloody, itchy, and ulcerated, and contain necrotic, caseous or calcified granules (12, 50, 51).

If the lesion originates from a pre-existing lesion (as is typically the case), the wound develops into: dry, wet and edematous forms. The dry lesion is a generally circular alopecic area covered by grayish scales. The wet lesion is associated with moderate discharge and hair agglutination, whilst the edematous lesion is hairy and does not have a regular shape (3–5 cm in diameter); it is characterized by oedema and tiny nodules.

**Figure 2:**
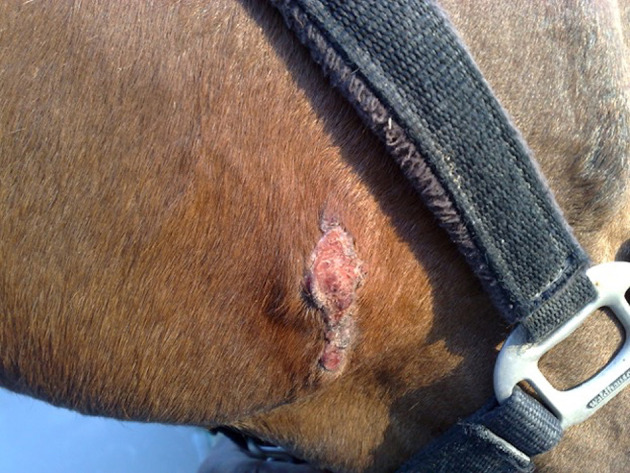
Healing process of a summer sore (original, A. Giangaspero).

**Figure 3:**
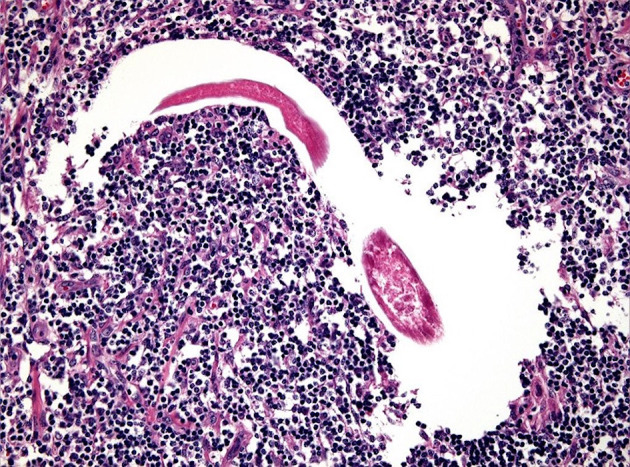
Numerous infiltrates of eosinophils and parasitic forms related to habronematids at histological exam (original, A. Petrella).

Lesions may heal (Figure 2) or recurrent lesions may evolve into non-healing granulomatous cancer-like masses; these may attract more flies, leading to a super-infection (9, 51, 52). Histologically, the wounds are infiltrated with eosinophils, macrophages, lymphocytes and a few plasmacells. In peripheral areas, an abundance of vascular and fibro-connective tissue can be observed, with masses of eosinophils in coagulation necrosis. Sections of nematodes can be also detected (53) (Figure 3).

In the *muco-cutaneous habronematidosis* conjunctiva, medial canthus, nasolacrimal ducts, or commissure of the lips or urethral process, glans, prepuce, vaginal fornix are involved. When larvae are released in the eyes or on the periocular tissue, typically in the medial canthus, infected animals present marked conjunctivitis, blepharitis, dermatitis with photophobia and lacrimation (6, 32, 54).

This form is only apparently uncommon; five cases of (peri)ocular habronematidosis have been recently published in the Netherlands (33) but we speculate that many other cases might have not been published. Affected horses may show profuse mucopurulent discharge, and from moderate to evident blepharospasm and/or epiphora. Some may suffer of ectropion and chemosis. Gross lesions are ulcerative and granulomatous with so-called sulfur-like granules within and around the lesion (from 5 mm to 1.5 cm in diameter up to 25 × 10 cm) that appear on the palpebral conjunctiva of the medial canthus (33, 55). The histological examination shows a marked infiltration of multifocal to coalescing eosinophilic granulomas, and a nucleus of eosinophilic necrotic debris together with many degenerate eosinophils delimited by epithelioid macrophages with few lymphocytes and plasma cells in the adjacent tissue, with occasionally multifocal moderate to large clusters of coccoid bacteria in most of the affected animals (33). When the prepuce, urethral process, vaginal fornix are affected, animals show dysuria and frequent urination due to the presence of different degrees of fibrosis (56, 57). Histological exam of the mucous membranes reveals granulation tissue, infiltrated by eosinophils and affected by collagenolytic phenomena (54).

The pathogenesis of the *pulmonary habronematidosis* is unclear, and how larvae reach the lungs is not fully understood (44, 46, 58). Larvae on skin wounds may move to the lungs via the bloodstream, or larvae released in the nostrils or mouth mucosa can reach the lungs via the trachea (59). In any case, the parasites damage the peribronchial tissue causing large nodules-−0.2–2 cm of diameter—which contain larvae or residues of larvae (42).

*Erratic forms* are described for *D. megastoma*; larvae belonging to this species can reach the brain and form small nodules (60).

## Diagnosis

Gastric habronematidosis cannot be easily diagnosed, because the unspecific clinical signs, that characterize this form, may easily be confused with other diseases. The detection of larvated eggs by flotation or—although less successfully—of larvae using the Baermann technique or coproculture has been widely performed. The xenodiagnosis is considered the gold standard even though it is challenging and time-consuming; in alternative, a modified Mertiolate-Formaldehyd-Concentration (MFC) technique has been proposed for suspected gastric forms by *H. muscae* (61). All these traditional techniques have a very low sensitivity, even if the parasitic burden is high.

Another diagnostic approach is gastric lavage combined with microscopic examination of the sedimented washings, but this method is invasive, dangerous and laborious; in addition, the patient has to be necessarily anesthetized and restrained.

Differental diagnosis of cutaneous and muco-cutaneous forms is also challenging, in fact, the clinical signs, the granulomatous lesions in particular, may overlap those of other diseases, i.e., botryomycosis, pythiosis, phycomycosis, onchocercosis, equine sarcoid, and squamous cell carcinoma (54, 62–65). Diagnosis may be even more challenging when coexistence of sarcoid and habronemosis occur (66). The surface of the lesion must be scratched in different areas and, in order to detect the larvae, the collected tissue has to be digested for 12–18 hours at 37°C in an acid pepsin solution (50). However, the larvae tend to be few, and might be digested or necrotic in the more chronic lesions. Also, they live for <1 month in cutaneous tissues, and larval death might cause even more necrosis and calcification than a living parasite (20).

Molecular diagnosis can be considered the gold standard. A semi-nested PCR was developed for detection and identification of *Habronema* (*H. microstoma* and *H. muscae*) DNA irrespective of their life cycle stages, with significant repercussions for clinicians. The PCR assay achieved a diagnostic specificity of 100% and a sensitivity of 97% (29, 36, 67, 68). This PCR was developed for the detection of habronematids in gastric form, but it was also able to detect *Habronema* DNA in skin samples from animals with summer sores (69). This can be considered a practical and beneficial approach for veterinarians for the diagnosis of both gastric and cutaneous habronemosis, which are sometimes hard to differentiate from other gastric and skin diseases of equids with comparable clinical signs (29).

## Prophylaxis and Treatment

As extensive husbandry conditions seem to be the most effective against habronemosis (70), prophylaxis to decrease the incidence and prevent the reappearance of habronemosis (32, 54, 65) are regular cleaning of the stables and paddocks with proper removal and disposal of manure as part of an integrated fly control plan. Against flies, horses can also be treated with licensed repellents (71) or protected mechanically (using fly nets and blankets); however, some animals do not tolerate fly masks.

Studies focusing on the efficacy of macrocyclic lactones against intestinal strongyles have shown the high efficacy (up to 100 per cent) of ivermectin (200 μg/kg) (72–74) or moxidectin (400 μg/kg) (72–75) against worms in the stomach (49, 65). A more recent study from Brazil demonstrated an efficacy of 92–95%, 98–100%, and 100% of ivermectin, abamectin, and moxidectin, respectively, against *H. muscae* (76). Despite being mostly side data, the efficacy of macrocyclic lactones against gastric *Habronema* was evident, as also confirmed by a focused study that ultimately proved the efficacy of moxidectin against *H. muscae* (77).

For *Habronema* lesions, a single dose of ivermectin did not provide solid evidence of efficacy (49). In some cases, the use of anthelminthic drugs is debatable; for instance, as in the periocular localization the lesions are thought to be a result of local hypersensitivity to dead or dying larvae, the administration of ivermectin may worsen the signs of pruritus (78).

Other drugs (ivermectin, echothiophate, and trichlorfon) have been described for treating cutaneous or mucocutaneous forms (33); in addition, corticosteroids were used to reduce inflammatory hypersensitivity reactions. These molecules can be used mostly in ocular habronematidosis and administered systemically, topically, intra-lesionally or sub-conjunctivally (33, 55).

Surgical debulking intervention is indicated when the medical treatment of summer sores is refractory.

## Conclusions and Future Perspectives

Only old-fashioned studies on gastric habronemosis are available in the literature and this would suggest a disappearance or significant reduction of this disease; however, the recent case-reports on cutaneous habronemosis demonstrate that it is maybe not the case.

Even with swinging academic or technical interests, in its whole the understanding of habronemosis has improved. However, several questions remain unanswered.

A productive multidisciplinary increasingly current research approach that manages the key zones of science, epizoology finding, and treatment, is expected to upgrade the information base and improve the counteraction and control of habronematidosis. Here some tips:

- *Prevalence*: Since clinical cases of cutaneous habronemosis are reported, data on prevalence of gastric habronemosis to which cutaneous habronemosis is related, need to be updated. The lack of information about *Habronema* prevalence is connected with diagnostic tools. Considering that common quali-quantitative copromicroscopic diagnostic approaches are not responsive enough for the detection of *Habronema* eggs and studies at necropsy are time-consuming and difficult to perform, molecular tools must be considered the “gold standard” for future works.
- It is remarkable that PCRs have been designed for the detection of *H. microstoma* and *H. muscae*, but not for *D. megastoma*. Once set up, it could be found out that this latter species is more distributed than expected.
- *Habronema microstoma* vs. *Habronema majus identity*: data on interspecific difference (using *ITS* and *cox1* genes) between *H. microstoma* and *H. majus* have been recently provided (4) but further analysis on the microscopic and genetic make-up of variations among individuals from various geographical areas are needed to ultimately confirm that *H. majus* is a separate species.
- *Host-vector relationship*: The role of *M. domestica* as vector of *H. microstoma* (40, 42) needs to be explored and in particular issues on the cellular, molecular, and/or immunological response of insects related to the possible species-specific susceptibility should be faced. Moreover, due to the different feeding behavior, the role of *S. calcitrans* in trasmitting *H. microstoma* should be further investigated.
- *Anthelminthic resistance*: currently, while there is evidence of increased resistance of the equine cyathostomins and *Parascaris equorum* to various anthelminthics (79), there are no studies on the resistance of *Habronema* species to anthelmintics because these parasites are not included in the standard fecal egg count reduction tests generally used in surveys documenting drug resistance, because their eggs are very difficult to detect with copromicroscopical concentration techniques. Improvements on diagnostic strategies (as above reported) may help to fill also this gap of knowledge.
- *Targeted and alternative treatment*: The widespread anthelmintic resistance calls for alternative control strategies, i.e., to develop novel non-chemicals. The recent *in vitro* experiment on anthelmintic properties of *Verbesina alternifolia* (crown beard) against *H. muscae* which demonstrated by SEM an irreversible degenerative change of the treated worm (80) may be a stimulus to work on this aspect in field condition.

## Author Contributions

AB and AG executed a first draft. DT and RP helped draft the manuscript. All authors contributed to the article and approved the submitted version.

## Conflict of Interest

The authors declare that the research was conducted in the absence of any commercial or financial relationships that could be construed as a potential conflict of interest.

## Footnotes

^1^Search strategy and selection criteria: The articles cited in this Review were searches by the PubMed, Scopus, and Google Scholar databases (up to April 10, 2020) and carried out using the terms “habronem^*^” AND (“taxonomy” OR “nomenclature”); “habronem^*^” AND (life-cycle” OR “transmission” OR “host”); “habronem^*^” AND (“histo^*^” OR “clinical manifestation” OR “syndrome”); “habronem ^*^” AND (“epidemiology” OR “epizootiology” OR “prevalence”); “habronem ^*^” AND (“horse” OR “donkey” OR “equid”); “habronem^*^” AND (“season”); “habronem^*^” AND (“detection” OR “diagnosis” OR “microscopy”); “habronem^*^” AND (“treatment” OR “chemotherapy”); “habronem^*^” AND (“immunol^*^”); “habronem^*^” AND (“prevention” OR “control”; “habronem^*^” AND (“molecular” OR “genom^*^”). The same searches were conducted using the term “draschi^*^” and “habronematid”. A total of 250 publications were identified; we screened the titles and abstracts and identified articles with relevant content and context. The full texts of these articles were read to verify their relevance to the present topics in this Review.

^2^Most authors consider *Habronema majus* and *Habronema microstoma* as the same species. Recent morphological findings related to the arrangement of the caudal papillae in the male and molecular (in *ITS2* and in *cox1*) interspecific differences between *H. majus* and *H. microstoma* from donkeys indicate that they could be separate species (4).
